# Treating corneal neovascularization using a combination of anti-VEGF injection and argon laser photocoagulation application - case report

**DOI:** 10.22336/rjo.2021.58

**Published:** 2021

**Authors:** Mariela Grossi Donato, Elias Donato, Marina Álvares de Campos Cordeiro, Matheus Martins de Andrade, João Alberto Holanda de Freitas

**Affiliations:** * Ophthalmology Resident, Instituto de Olhos Ciências Médicas, Belo Horizonte, Minas Gerais, Brazil; ** Department of Ophthalmology, Ocular Medical Center - Belo Horizonte, Minas Gerais, Brazil; *** Department of Ophthalmology, Holanda Oftalmologia – São Paulo, Minas Gerais, Brazil

**Keywords:** corneal neovascularization, anti-VEGF

## Abstract

The present study aimed to demonstrate the possibility to treat corneal neovascularization using the combination of anti-VEGF injection and argon laser photocoagulation.

## Introduction

Since the first antiangiogenic drug (AGF) launched in 2004, called Macugen (pagaptamid), Baush and Lomb and others have succeeded in promising efficiency, all aimed at treating Age-Related Macular Degeneration (AMD). We referred to the following: Avastin (bevacizumab) Roche; Lucentis (ranibizumab) Novartis; Eylia (aflibercept) Bayer. Later we started to observe that its application would solve cases of neovascularization in the anterior segment, such as rubeosis iridis, etc.

The purpose of this report was to show that the injection of AGF in association with the argon laser could inhibit corneal neovascularization secondary to some inflammatory process.

Among its various functions, the cornea plays a fundamental role in the transmission and refraction of light that falls on the eyes. For an adequate process of light transmission to the photoreceptors of the retina, the cornea must be transparent, aiming at the formation of the image [**1**].

This process of corneal transparency is called “Corneal angiogenic privilege (lymph)”, and, in the absence of pathology, it can inhibit the formation of blood and lymph vessels, which can cause corneal opacity and prevent the physiological process of passing light rays [**2**]. This “privilege” consists of a balance between inhibitory and stimulating factors for angiogenesis in the corneal epithelium; and an imbalance in this process, caused by an inappropriate local tissue reaction secondary to several factors, including infections, traumas, and immunological reactions, can induce corneal neovascularization (NVK) [**3**]. Neovessels present structural changes such as the absence of pericytes and basement membrane, which allow fluid, proteins, and lipids to leak into the extracellular space. This culminates in edema and infiltrates, which reduce the transparency of the cornea and, consequently, visual acuity. In addition, neovessels favor the rejection of corneal transplantation due to the easy access of antigen-presenting cells to donor tissue [**4**]. NVK is the leading cause of corneal blindness in the world (trachoma) and in industrialized countries (herpetic keratitis) [**5**].

## Case report

CGS, 68, complained of low visual acuity (LVA) in both eyes (BA) at the first consultation on 01/22/2019. She reported past ocular herpes and denied concomitant pathologies. On clinical examination, she had uncorrected visual acuity (UVA) right eye (RE) 20/ 50, left eye (LE) 20/ 200. Dynamic refraction in RE: -1.00 -0.50 165 20/ 30; LE without vision improvement with correction. Fundoscopy (FO) DO unchanged, but not viable in LE. Tonometry RE 11 mmHg OE 13 mmHg.

**Fig. 1:**
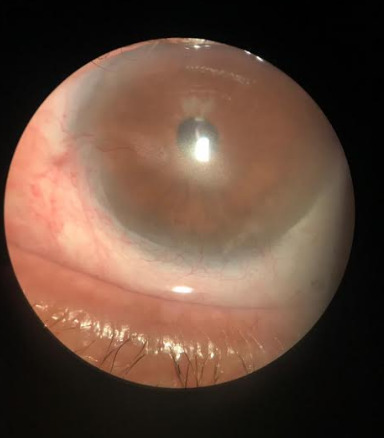
1st day: neovascularization with branches from the periphery to the center, paracentral leukoma, and stromal infiltrate in the central area of the cornea (Reference: Personal file of Dr. Elias Donato)

Biomicroscopy diagnosed grade II nuclear cataract in RE and pseudophakia in LE. LE also presented neovascularization with branches from the periphery to the center, in addition to paracentral leukoma and stromal infiltration in the central area of the cornea (**Fig. 1**).

Phacoemulsification was indicated with intraocular lens implantation in RE. In LE, we opted for treatment of the corneal condition using photocoagulation with an argon laser (PAL), associated with antiangiogenic therapy (AVGF) and topical corticotherapy.

**Fig. 2:**
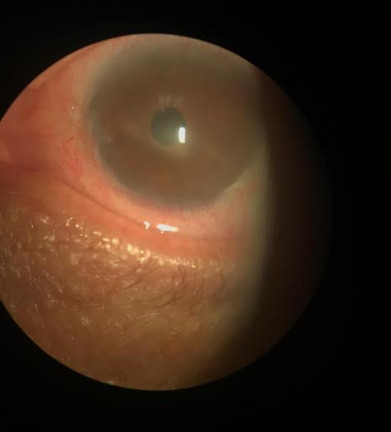
Cornea after the first session of FCG associated with antiangiogenic (02/06/2019). Significant reduction in neovascularization and stromal infiltrate with the persistence of some new vessels (Reference: Personal file of Dr. Elias Donato)

**Fig. 3:**
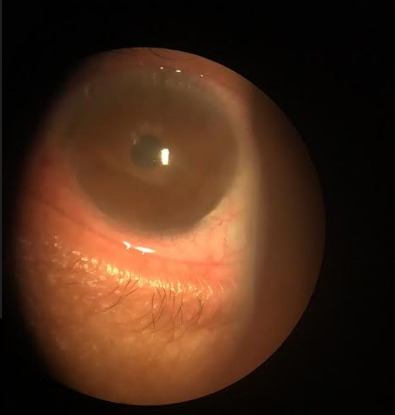
Cornea after the second session of FCG associated with antiangiogenic (03/26/2019). The almost total reduction of the vascular pannus and stromal infiltrate (Reference: Personal file of Dr. Elias Donato)

**Fig. 4:**
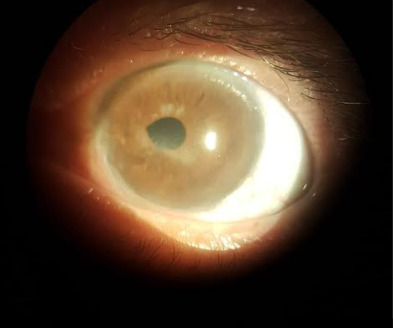
Stromal infiltrate (Reference: Personal file of Dr. Elias Donato)

**Fig. 5:**
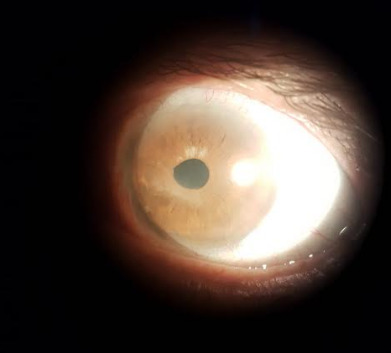
Cornea after the third session of FCG associated with antiangiogenic (04/30/2019). Total regression of the vascular and infiltrated pannus, maintaining a discrete paracentral opacity (Reference: Personal file of Dr. Elias Donato)

## Objective

The objective of the present work was to report the results obtained in the treatment of corneal neovascularization, using the concomitant therapy of photocoagulation with an argon laser and AVGF.

## Methods

The patient was prepared with topical anesthesia in LE and submitted to the first session of FGC on 01/30/2019, in which 35 shots were applied at 250 mW, 400 microns, in continuous mode, in addition to the subconjunctival and intra-stromal perilimbal injection of 0.15 ml (3.75 mg) of bevacizumab (Avastin®, Roche AG, Basel - Switzerland). Topical fluorometholone acetate was also started in 4 daily applications.

On 02/06/2019 the patient returned, reporting a slight improvement in visual acuity in LE. Biomicroscopic examination revealed a significant reduction in neovascularization and stromal infiltrate. Some neovessels persisted and a new PAL session was held on the same day, during which 25 shots were fired at 200 mW, 400 microns in continuous mode (**Fig. 2**).

On 03/26/2019, the patient returned, reporting an important improvement in subjective visual acuity. The exam showed an almost total reduction of the vascular pannus and stromal infiltrate. Visual acuity improved significantly, reaching 20/ 40 with correction (**Fig. 3**).

On 03/27/2019, a new FGC session was held with 30 shots at 200 Mw, 400 microns in continuous mode, and a new perilimbal injection of bevacizumab. The latter was the same dosage as the first performed. PAL was repeated on 03/03/2019 with 45 shots, 200 mW, 200 microns in continuous mode (**Fig. 4**).

During the examination on April 30, the patient presented total regression of the vascular and infiltrated pannus, maintaining a discrete paracentral opacity and an AV 20/ 40 with correction (**Fig. 5**).

## Results

Upon completion of the proposed therapy, the patient presented an important regression of the corneal neovascularization with the elimination of the paracentral vessels and an important reduction of the stromal infiltrate. The final visual acuity was equal to 20/ 40 in LE +0.5 -0.50 x90.

## Discussion

The pathogenesis of NVK begins with a precursor lesion, which precipitates dilation of the prelimbic vessels, followed by recruitment of leukocytes and release of growth factors that stimulate the increase in vascular permeability of the endothelium and degradation of the extracellular matrix, enabling the migration of these cells to the cornea, where the new vessels will form [**4**].

NVK can have different etiologies. Among them are the bacterial infections such as Chlamydia trachomatis, Staphylococcus spp., Pseudomonas, Treponema pallidum, among others. Viruses like herpes and fungal agents like Candida and Aspergillus, as well as autoimmune pathologies like Sjogren, Terrien’s marginal degeneration, and pemphigoids [**2**] are possible causes. Burns with acids and alkalis, as well as the use of contact lenses are also among possible causes [**6**].

There is a strong expression of vascular endothelial growth factor (VEGF) in neovascularized corneas, which also occurs in other ocular pathologies such as age-related macular degeneration and diabetic retinopathy. AVEGF has been used as a treatment for these diseases since 2005 and is safe for ocular use, including for most recent corneal neovascular pathologies [**4**].

The choice of appropriate treatment for NVK can be challenging, and several models have been tested for the various models of NVK [**1**]. In addition to treating the underlying cause, the treatment of NVK involves the destruction of the new vessels and restoration of the zone of angiogenic privilege, preventing a new neovascular process. Therefore, the approaches can be classified into three categories: anti-angiogenic, angio-regressive, and angio-occlusive [**7**].

Anti-angiogenic agents (anti-VEGF) can be administered by topical ocular, subconjunctival, intrastromal, and intravitreal agents. Bevacizumab (Avastin; Roche) is a recombinant humanized monoclonal antibody that recognizes all forms of VEGF and inhibits the VEGF/ VEGF-R interaction. It was initially approved by the FDA for the treatment of metastatic colorectal carcinoma but is effective in AMD and several studies have corroborated its usefulness in NVK. Ranibizumab (Luscentis; Roche) is produced from the Fab fragment of the same antibody used to produce bevacizumab and binds more strongly to VEGF-A [**9**]. These compounds have also been shown to be effective in inhibiting lymphangiogenesis, both from the direct action on the lymphatic epithelium, as well as indirect on the release of macrophages, which is a promise in their use to inhibit graft rejection [**7**]. Despite the antiangiogenic action, anti-VEGF can cause adversities such as delay in endothelial recovery and decreased corneal thickness due to the neurotrophic effect of VEGF [**10**]. In this sense, studies are still needed to clarify drug efficacy, dosage, and safety.

Photocoagulation (FCG) with argon laser is an angio-occlusive component, usually applied in association with bevacizumab; studies show that the drug’s effect is greater when simultaneously applied to chemical cauterization [**4**]. PAL is a safe method, but it can cause complications related to inflammatory overload, in addition to causing iris atrophy, pupil ectasia, and hemorrhages [**8**].

## Conclusion

The aim of the article was to evaluate the effect of AVEGF associated with PAL in the reduction of corneal neovascularization. The decrease in neovessels in the cornea proved that the combination influences the improvement of the transparency of the cornea and, consequently, the visual acuity; thus, improving the prognosis and a possible corneal transplant.

**Conflict of interest**

The authors declare no conflicts of interest.

**Informed Consent and Human and Animal Rights statement**

Informed consent has been obtained from all individuals included in this study.

**Authorization for the use of human subjects**

Ethical approval: The research related to human use complies with all the relevant national regulations, institutional policies, is in accordance with the tenets of the Helsinki Declaration, and has been approved by the Ethics Committee of Ciências Médicas College, Belo Horizonte/MG, Brazil.

**Acknowledgements**

None.

**Sources of funding**

The authors declare that their research has not been funded by any entity. All authors had no financial disclosures or support in this work.

**Disclosures**

None.

## References

[1] Ellenberg D, Azar DT, Hallak JA, Tobaigy F, Han KY, Jain S (2010). Novel aspects of corneal angiogenic and Lymphangiogenic privilege. Progress in retinal and eye research.

[2] Bock F, Maruyama K, Regenfuss B, Hos D, Steven P, Heindl L (2013). Novel anti (lymph) angiogenic treatment strategies for corneal and ocular surface diseases. Progress in retinal and eye research.

[3] Menzel-Severing J (2012). Emerging techniques to treat corneal neovascularisation. Eye (London, England).

[4] Mello GHR, Lupion FG, Oliveira FM, Bude LR, Wasileweski D, Cavalcati TC, Moreira H (2011). Ação do bevacizumabesubcojuntival na neovascularização e re-epitelizaçãocorneana 25 dias após queimadura química. ArqBras Oftalmol.

[5] Stevenson W, Cheng SF, Dastejerdi MH, Ferrari G, Dana R (2012). Corneal neovascularization and the utility of topical VEGF inhibition: ranibizumab (Lucentis) vs. bevacizumab (Avastin). The Ocular Surface.

[6] Lee P, Wang CC, Adamis AP (1998). Ocular neovascularization: an epidemiologic review. Survey of Ophthalmology.

[7] Rodrigues M (2015). Neovascularização da córnea – perda de um privilégio (214-2015). Tese (mestrado integrado em medicina). Faculdade de Medicina Universidade do Porto.

[8] Dantas PE, Dantas MC, Holzchuh N, Celis VFH (1997). Argon e YAG laser no tratamento de neovascularização de córnea. Arq, Bras. Oftal.

[9] Chang JH, Garg NK, Lunde E, Han KY, Jain S, Azar DT (2012). Corneal neovascularization: an anti-VEGF therapy review. Survey of Ophthalmology.

[10] Bock F, Onderka J, Rummelt C, Dietrich T, Bachmann B, Kruse FE (2009). Safety profile of topical VEGF neutralization at the cornea. Investigative Ophthalmology & Visual Science.

